# Application of QCM in Peptide and Protein-Based Drug Product Development

**DOI:** 10.3390/molecules25173950

**Published:** 2020-08-29

**Authors:** Dorian Migoń, Tomasz Wasilewski, Dariusz Suchy

**Affiliations:** 1 Department of Inorganic Chemistry, Faculty of Pharmacy, Medical University of Gdańsk, Hallera 107, 80-416 Gdańsk, Poland; tomasz.wasilewski@gumed.edu.pl; 2 Polpharma Biologics S.A., Trzy Lipy 3, 80-172 Gdańsk, Poland; dariusz.suchy@polpharmabiologics.com

**Keywords:** AT-cut crystals, quartz crystal microbalance, protein formulation, sensors, biosensors, peptide aggregation, protein aggregation

## Abstract

AT-cut quartz crystals vibrating in the thickness-shear mode (TSM), especially quartz crystal resonators (QCRs), are well known as very efficient mass sensitive systems because of their sensitivity, accuracy, and biofunctionalization capacity. They are highly reliable in the measurement of the mass of deposited samples, in both gas and liquid matrices. Moreover, they offer real-time monitoring, as well as relatively low production and operation costs. These features make mass sensitive systems applicable in a wide range of different applications, including studies on protein and peptide primary packaging, formulation, and drug product manufacturing process development. This review summarizes the information on some particular implementations of quartz crystal microbalance (QCM) instruments in protein and peptide drug product development as well as their future prospects.

## 1. Introduction

Biologics are currently one of the fastest growing sectors of the pharmaceutical industry [1]. Specifically, this group of modern and dynamically developing therapeutics constituted the majority of best-selling drugs in 2019 [2]. Moreover, patent protection and exclusivity rights of many biological medicines either have expired or will expire in the near future. Hence, a significant interest in the production of their cheaper, off-patent counterparts, biosimilars, has been observed among pharmaceutical companies. The biosimilar market, as an additional part of biologics sector, will contribute to health care costs reduction and, thus, to an increase in access to innovative therapies for patients [3]. One of the largest groups of biologicals are recombinant DNA-derived proteins and peptides. Development of drug products based on the abovementioned molecules is challenging and at the same time burdened by a high failure risk. This results from the fact that both proteins and peptides, because of their structural complexity, are characterized by an enhanced susceptibility to various environmental factors [4].

Generally, two main degradation pathways of peptides and proteins can be distinguished, (i) chemical degradation (i.e., covalent-bonded aggregation, disulfide exchange, deamidation, isomerization, racemization, fragmentation, oxidation, β-elimination, Maillard reaction, and diketopiperazine formation) and (ii) physical degradation (i.e., denaturation, unfolding, adsorption, and non-covalent aggregation) [5]. These instability events can occur during manufacturing, storage, handling, and administration to the patient [6]. Aggregation, as one of the physical degradation pathways in both protein and peptide drug products, has raised great concern among biopharmaceutical companies, academia, and regulatory agencies. This is due to the potential impact of some aggregates on pharmacokinetics, bioactivity and, most importantly, immunogenicity [7]. To date, many specialized techniques aiming at aggregate qualitative and quantitative evaluation, as well as aggregation propensity of products have been reported [8]. One of these, quartz crystal microbalance (QCM), a mass sensing method characterized by high resolution and sensitivity, offers an interesting, adsorption-based predictive tool utilizable in protein and peptide drug product development [9].

**Figure 1:**
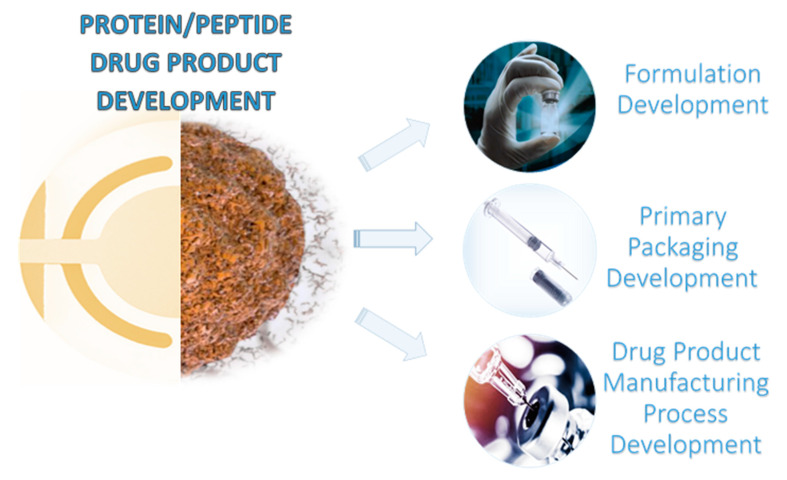
Key research findings of quartz crystal microbalance (QCM) applications in peptide- and protein-based drug product development.

This review is focused on the up-to-date applications of QCM equipment in the biopharmaceutical industry with particular reference to its applicability in formulation, primary packaging, and drug product manufacturing process development (Figure 1). In addition, technology basics, as well as peptide and protein adsorption mechanisms on the QCM surface, were also accounted for.

## 2. QCM Technology Basics

**Figure 2:**
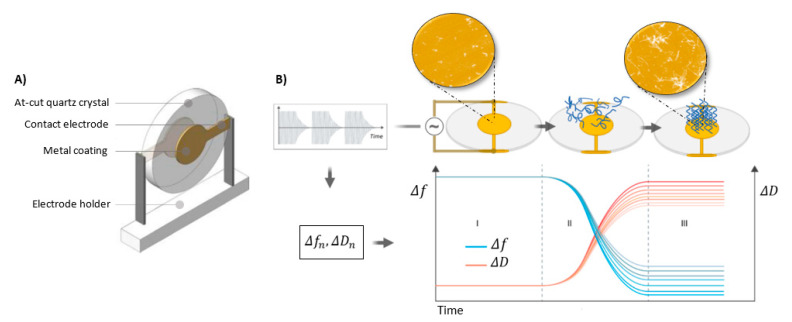
(**A**) Basic scheme of QCM sensor consisting of a piezoelectric AT-cut quartz crystal coated with two gold electrodes, one on each side. (**B**) Schematic working principle provides information on variations in *Δf* and *ΔD* plotted as molecules become adsorbed on gold sensor surface. In the schematic adsorption mechanism, section I shows a bare surface and stable baselines; during adsorption molecular changes in *Δf* and *ΔD* are observed (section II). After complete adsorption on the surface, the baselines are stabilized (section III). The collected quartz crystal microbalance with dissipation monitoring (QCM-D) data can be used for viscoelastic modelling and quantification of mass, as well as calculating the viscoelasticity and thickness of the adsorbed layer.

The recent development of microsystems and nanotechnology triggered significant progress in biological and biochemical research. Sensors such as QCMs have widely been applied to monitor interactions between biomolecules, for instance protein and peptide aggregation. QCM design is based on a piezoelectric quartz crystal placed between two electrodes (Figure 2). After connecting the electrodes to an oscillator and application of alternating voltage, the quartz crystal oscillates with a very stable resonant frequency (the so-called piezoelectric effect) [10]. QCM devices are simply designed and easy to use analytical instruments. However, they require fundamental knowledge about design and interpretation of generated data. The quartz crystal microbalance provides real-time mass change measurement owing to the application of a quartz resonator. The QCM transducers’ surfaces can be covered with different layers, such as metals, metal alloys, metal oxides [11], semiconductors, polymers, biomaterials, etc. [12,13,14]. The sensitivity of QCMs is about 100 times higher than that of a typical precise analytical balance, thus enabling observation of mass change at a nanogram level (for a typical piezoelectric sensor with 10 MHz frequency, a change in mass of 4.4 ng·cm^−2^ results in a frequency change of around 1 Hz) [15], as well as distinguishing single layers or even atomic monolayers [16]. If a thin, non-dispersible layer is uniformly deposited on an active electrode, the resonant frequency of the electrode decreases proportionally to the mass of the adsorbed layer. A relationship between frequency change and the adsorbed mass efficiency is expressed by the Sauerbrey equation [17] (1):

(1) $$\Delta f=-\frac{2f_{0}^{2}}{A\;\sqrt{\rho_{q}{µ}_{q}}}\Delta m$$

where, $\rho_{q}$ and ${µ}_{q}$ are the density (2.648 g·cm^−3^) and shear modulus of quartz (2.947 × 10^11^ g·cm^−1^·s^2^), respectively, $f_{0}$ is the unloaded crystal frequency, A is the crystal piezoelectrically active geometrical area, defined by the area of the deposited metallic film on the crystal, Δm and Δf are the mass and system frequency changes. According to this equation, the mass of a thin layer deposited on a surface can be calculated by measuring changes in the resonant frequency.

Due to the application of selected transduction techniques in chemo- and bio-sensors [18,19,20,21,22,23], it is possible to evaluate the deposition degree of particular materials [24], for instance in the form of self-assembled monolayers (SAM) [12,25], and to analyze gas phase composition [14,26,27]. Nowadays, the priority in design and development of QCM analytical platforms is given to naturally occurring compounds, since they are excellent receptors with superior selectivity for the target analytes [28]. The data acquired from liquid analysis require assessment of potential contributions from surface microporosity changes, which can be solved using a quartz crystal microbalance with dissipation monitoring (QCM-D), preferred in the measurement of molecular aggregation [29,30]. QCM-D technology enables measurement of not only frequency but also the dissipated energy of the oscillating crystal, which makes it a promising tool for investigation of the adsorption of biomolecules that have a tendency to form films with high viscoelasticity [31]. Upon contact with liquids, the crystal enables acquisition of the information about their density ($\rho_{L}$) and viscosity (η) via frequency change following the Kanazawa–Gordon Equation (2) [19]. If one side of the crystal is immersed in liquid, free resonant frequency accounts for additional shifts due to viscous damping from the liquid (2,3):

(2) $$\Delta f=-f_{0}^{\left(\frac{3}{2}\right)}\;\sqrt{\frac{\rho_{L}\eta }{\pi \rho_{q}\mu_{q}}}$$

(3) $$\delta =\frac{2\eta }{\omega \rho_{L}}$$

where, $f_{0}$ is the unloaded crystal frequency, $\rho_{q}$ is density of quartz, ${µ}_{q}$ is shear modulus of quartz, $\rho_{L}$ and η are the liquid density and viscosity, respectively. Equation (3) describes decay characteristic length (δ) proportional to the viscosity/density ratio of the liquid and as inversely proportional to the angular frequency (ω). For a 10 MHz AT-cut quartz crystal in contact with water of δ = 178 nm, the acoustic wave generated by the crystal and transferred to the liquid is completely damped at 178 nm on the crystal’s surface. Consequently, the quartz resonators react to a liquid, occurring in the direct vicinity of their surface. In the QCM-D method, a dissipation coefficient *(D)* is obtained via decay in crystal oscillation after a short perturbation impulse close to the resonant frequency (F_0_). The degree of protein immobilization on the crystal’s surface can be followed by monitoring the frequency changes, matching the obtained data to the Sauerbrey Equation (1). However, with samples containing water, just as with those for investigation of protein aggregation, coefficient D is a fundamental value for the acquisition of the full characteristics of the adsorbed structure of the deposited layer [32]. For energy dissipation D = Q^−1^, which is a reciprocal of oscillator quality coefficient, the following relation is obeyed (4):

(4) $$\Delta D=Q^{-1}=\frac{\Gamma }{\omega }=\frac{\sqrt{2\omega_{0}}}{\pi }\sqrt{\frac{\rho_{L}\eta }{\rho \rho_{q}\mu_{q}}}$$

where Γ is the bandwidth of the corresponding frequency, $\rho_{L}$ and η are the density and viscosity of the liquid on the crystal, respectively. Moreover, the shifts in frequency due to adsorption of the molecules from solution depend not on dry mass but on hydrodynamic mass including adsorbed molecules in water. More complex contributions to the frequency shift result from internal viscoelasticity and from changes of surface roughness upon aggregates’ growth [33].

A basic scheme of QCM construction and working principles are displayed in Figure 2. 

In practice, variations of the frequency and sensitivity are distributed and assume the shape of a Gaussian curve because of an energetic trap of the quartz resonator. This effect is not uniform over the entire surface [34]. To provide normal oscillations of the QCM, it is necessary to apply a metal layer on both sides of the quartz plate. QCM mass sensitivity can vary significantly depending on material, shape, thickness, and size of the metal electrodes. The possibility of using the quartz resonators in an aqueous environment opened new research prospects and widened biotechnological and biomedical applications. The reason, among others, was that biological samples usually call for a liquid medium to maintain their properties. The use of QCMs encompasses a wide range of sensors and biosensors for mass, viscosity, temperature, and humidity measurements, which can operate in both the gas and liquid phases [35]. Implementations in medicine and environmental monitoring are currently two fields, which benefit from the QCM technique owing to its reliability, high sensitivity, and ability to deposit a wide spectrum of materials. An important feature is also the short response time providing real-time results [36]. The QCM has been successfully utilized for diagnosis of diseases such as cancer [21,37,38,39] as well as viral [40] and bacterial [41] infections. Moreover, the QCM technique, especially QCM-D, is widely used in cellular characterization [42] and interactions [43,44,45], DNA detection [40,46], and protein detection [47], as well as high-throughput real-time screening of protein drug targets [48,49,50]. Overall, the QCM technology attracts a lot of attention due to the wide variety of possible applications [48,51,52,53]. The present review is mainly focused on the latest QCM instruments’ achievements in protein and peptide drug product development, with general operations reduced to basic concepts. Detailed developments and properties of QCM instruments have already been extensively presented in the literature [35,54,55].

## 3. Adsorption of Peptide and Protein Molecules on the QCM Surface

Recent years have witnessed a renaissance of QCM technology for the investigation of adsorption phenomena in biological systems. Kinetics of protein and peptide adsorption as well as the interactions with particular ligands are still subjects of interest [56]. QCM technology enables the monitoring of changes of deposited layers’ properties and of receptors binding with small and big ligands [22,57,58].

Surface investigations are aimed at, inter alia, learning how proteins adsorb onto the surface and what the consequences are of surface interactions. Protein adsorption is a key factor in cell activity, for instance in cell attachment, surface migration, proliferation, and differentiation of growth. Investigation of the processes associated with adsorption of biomolecules enables control of the adsorption phenomena and achieving desired structural-chemical properties. Adsorption control is crucial for the design of biomedical implants, development of biosensors, and elaboration of the materials, which force or limit biomolecular and cell adhesion, e.g., surfaces designed to reduce inflammatory responses [59,60,61]. Extending QCM technology to the processes of peptide and protein adsorption opened new perspectives regarding the explanation of their fundamentals. The molecular structure determined by an amino acid sequence has a substantial impact on the properties and activity of peptides and proteins. Apart from quantitative analysis of adsorbed biopolymers, QCM also allows determination of relative surface adsorption affinity, i.e., hydrophobic polystyrene versus hydrophilic silica [62] or graphene derivatives [52]_._

Peptides and proteins play various roles in the human organism, which are driven mainly by amino acid sequence and native conformation. They also reveal a distinct tendency and susceptibility to aggregation and amyloid formation. These processes underlie pathological events associated with a wide range of disorders. Aggregation is also a serious problem in the field of biotechnology and pharmacy where it interferes with the characteristics of therapeutically active peptides and proteins [63,64]. Excessive deposition of insoluble, amorphous, and ordered protein or peptide aggregates in human organs and tissues leads to serious biological dysfunctions. Moreover, aggregation of drugs can drastically affect their bioavailability and storage possibilities, decreasing their availability and therapeutic effectiveness. Unfortunately, efficient methods of complete aggregation inhibition are not currently widely available. 

Protein folding is one of the most important physiological processes and on the other hand it is associated with pathological states known as conformational diseases or amyloidosis. Amyloids are highly ordered, fibril-aggregated structures and, independently of a protein precursor, they are characterized by a similar morphology and high thermodynamic stability. It should be emphasized that many proteins not connected with any diseases undergo in vitro fibrillation upon partial denaturation conditions [65]. During the aggregation process, native proteins undergo incorrect folding, which leads to the formation of insoluble amyloid fibrils having β-sheet structure (peptide chains oriented in parallel and anti-parallel ways) [66]. The amyloid fibrils might be cytotoxic and their toxicity is correlated with the fibrils’ morphology [67]. Oligomeric pre-fibril structures are currently believed to be the most toxic [68]. However, recent investigations also suggest existence of non-toxic oligomeric species [69].

Despite significant progress in both amyloid formation analysis, as well as general protein aggregates’ characterization [70,71] there is still a need for the results of quantitative in vitro measurements of the kinetics and thermodynamics of aggregates’ growth. They are necessary to elaborate a systematic approach to investigation of the strategies aimed at prevention or delay of aggregates’ formation onset and the characterization of the fundamental parameters conditioning the aggregation processes. In vitro as well as in vivo aggregation processes are the routes to incorrect folding of the native proteins. The most important factors influencing the aggregation process are hydrophobicity of side chains of amino acids and interactions between aromatic residues [72]. Physicochemical conditions, namely temperature, high or low pH, and oxidizing agents also determine aggregation. The stabilization of mature protein fibers depends on protein concentration and presence of steric effects in an amino acid sequence [73]. Interest in the amyloid form of peptides and proteins grew drastically during the last decade, mainly because of the classification of pathological amyloid fibrils as the precursors of many neurodegenerative diseases. To date, more than 40 diseases in humans, including Alzheimer’s, Parkinson’s, Huntington’s, and prion diseases, non-neuropathic systemic amyloidosis, and various non-neuropathic localized diseases, such as cataract and type 2 diabetes mellitus [74], have been associated with aggregation of (partially) unfolded peptides and proteins into cytotoxic amyloid aggregates via the formation of intermolecular β-sheets [75]. They are also likely to play a key role in numerous biological processes occurring in different organisms, e.g., human premelanosome amyloids can sequester highly reactive oxidative intermediates generated from melanin synthesis, and thereby protect melanocytes from oxidative damage [76]. The upsurge of interest in the amyloid structures of peptides and proteins related to health disorders (Table 1) is associated with increasing costs of medical care and difficulties in contemporary societies [77].

Studies performed so far have shown that despite the diversity of amino acid sequences of peptides and proteins, the aggregation process occurs according to a similar mechanism. It is believed that initiation of the aggregation process is related to short amino acid sequences (protein segments), built mainly of hydrophobic amino acid residues, self-recognition elements (SRE), and amyloidogenic hot spots. Short peptide fragments (di-, tri-, tetra-peptides) act as precursors of aggregation of whole proteins under in vitro conditions; these are often SRE fragments triggering inhibition of the native protein aggregation process [88].

A decisive influence on promotion and inhibition of amyloid aggregation originates from physicochemical characteristics of the surface such as hydrophobicity or charge because they have a direct impact on an interaction with monomers. Slight changes of these properties can affect the conformation and mobility of the adsorbed monomers. They can also influence the interaction of proteins and peptides and thus modulate their aggregation [89]. The types of amyloidogenic interactions of peptides and proteins with model surfaces are currently topics of intensive research [90]. A number of techniques were elaborated to estimate kinetic aspects of the aggregation encompassing monitoring the increase in light scattering of a growing amyloid suspension or the change in fluorescence or absorption of dyes that bind to amyloid [91,92]. However, heterogeneity of formed aggregation products and lack of detailed knowledge about their structure at the molecular level make quantitative interpretation troublesome in certain cases. Modern techniques enabling observation of fiber growth include atomic force microscopy (AFM), total internal reflection fluorescence microscopy (TIRFM), experiments that detect the growth of an ensemble of fibrils by surface plasmon resonance (SPR) or dynamic light scattering (DLS). Moreover, there are also techniques employing mass spectrometry (MS) coupled with high-performance liquid chromatography (HPLC) that monitor variations in concentration of the precursor protein as a determinant of incorporation during the fibers’ growth process [93,94]. An alternative approach to monitoring amyloid formation, enabling, among others, measurement of the kinetics of amyloid growth via real-time monitoring of increase in fiber mass, is based on QCM technology. Its implementation provides precise information about monitored desorption and adsorption processes, as does the SPR technique [95]. This technique is widely utilized for investigation of the aggregation process as well as identification and characterization of inhibitors and modulators of that process. Evaluation of the rate of fibril growth on different surfaces is currently widely examined [96,97], as well as the possibility of QCM-D application to study amyloid aggregation upon contact with various model surfaces, for example double lipid layers, self-assembled monolayers (SAMs), and metal oxides [25,83,98,99,100]. The benefits stemming from monitoring of dispersion changes include the option of discrimination between the types of amyloid adsorption [85] and characteristic fibril growth and conformations [25]. Formation of the fibrils often generates complex sensors response requiring application of appropriate sensor models and supplementary microscopic measurements, e.g., ex-situ AFM, fluorescence microscopy [25], or total internal reflection fluorescence (TIRF) [101]. One of the latest achievements is the elaboration of a combined QCM-TIRF technique allowing simultaneous measurement of the mass of a peptide adsorbed on the sensor’s surface and visualization of fibril growth using the TIRF microscope [24]. Performance, stability, and reliability of QCM biosensors have been limited by steps required for functionalization and activation of their surfaces, causing increase in film thickness and reduced biological activity. Kabay et al. [102] eliminated chemical steps by introducing a sensing layer modification using electrospun amyloid-like bovine serum albumin nanofibers on QCM surfaces. This modification enables the direct immobilization of bioactive agents by eliminating any surface functionalization process for further mass-sensitive biosensor applications. Understanding the molecular mechanisms of aggregation should provide a better comprehension of the interaction of aggregates with cell membranes and should contribute to the elaboration of new, improved therapies for diseases caused by protein and peptide aggregation.

**Figure 3:**
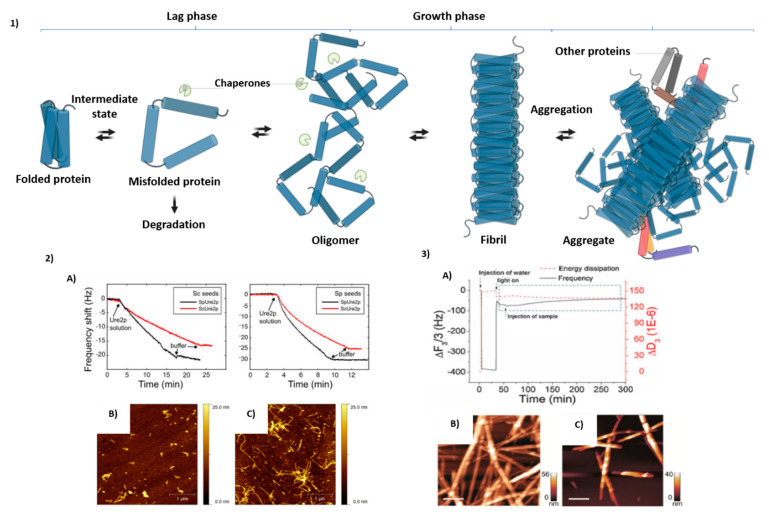
(**1**) Mechanism of amyloid formation proposed by Stroo et al. [103]. Misfolded protein can be refolded, degraded, and aggregated. The first step in the aggregation pathways involves oligomers, followed by fibril formation around the fibril axis and aggregation. (**2**) Measurement of fibril growth kinetics using QCM (2A) and atomic force microscopy (AFM) (2B,C) performed by Wang et al. [78]. (**3**) Utilization of QCM for evaluation of the degradation of amylin (20–29) aggregated by Au/g-C_3_N_4_ under light irradiation. (3A) AFM images of amylin (20–29) aggregates and images after photodegradation (3B,C) [79].

Protein aggregation is a complex process affected by many factors, pathways, and mechanisms. Under appropriate conditions any protein could form amyloid-like structures with their possible degradation. Schematic representation of those mechanisms is presented in Figure 3 accompanied by possible monitoring by QCM techniques. 

## 4. Application of the QCM in Formulation and Primary Packaging Development

Almost all of the currently commercially available biologics and peptide drugs are offered either as liquids or lyophilizates for parenteral use [104]. Degradation of those drug molecules is likely to occur during their production, processing, storage, and administration. It is common for both liquid and solid presentations and depends on, inter alia, amino acid sequence, isoelectric point, and hydrophobicity of the molecule as well as storage temperature, container, and composition of the drug product [6]. The degradation products can reduce drug activity and, in the worst case, enhance its immunogenicity. Moreover, a strong immune response can lead not only to neutralization of medicine, but also of other, endogenous proteins [105]. Thus, the main purpose of therapeutic formulation is to assure the protein and peptide stability so that the drug product remains safe for the patient [5,6]. 

As mentioned above, many of the degradation routes are highly dependent on the pH and ionic strength of solution (composition), as well as the primary packaging (storage container). Although it is virtually impossible to prevent all degradation pathways from occurring, development of an effective formulation might minimize it to an acceptable extent [106]. Two of the most important peptide and protein degradation pathways are aggregation and adsorption. The QCM is an interesting, sensitive technique which can be utilized for testing those phenomena. In addition, it can be applied to study the potential interactions between drug molecule and excipient. So far, several articles describing the application of the QCM technology for drug product development have been published. 

For instance, Härtl et al. [107] applied the QCM system to study stabilizing effectiveness of hydroxypropyl β-cyclodextrin (HPβCD) in monoclonal antibody solution subjected to shaking stress. Both the unfolded and native form of IgG antibodies exhibited a reduced tendency to undergo adsorption on a gold QCM electrode in the presence of HPβCD. The authors explained this phenomenon as a result of protein-cyclodextrin complex formation, which was characterized by a reduced hydrophobicity as compared to lone protein. 

Another example of application of QCM technology in drug product development is a series of research articles dedicated to protein-silicone oil interactions. Silicone oil is widely used as a lubricant in prefilled syringes and cartridges to ensure functional and consistent drug delivery [108]. Moreover, in some cases vials are also internally siliconized to reduce the incident of molecule adsorption to the glass surface [5] or enable a free drainage of the solution [109]. On the other hand, silicone oil may induce aggregation of certain proteins and peptides [110,111]. Therefore, it is essential to evaluate protein adsorption kinetics at the oil/water interface, as well as the influence of excipients on the adsorption. Dixit et al. [112] utilized the QCM to evaluate adsorption of Fc-fusion protein to silicon oil-coated crystal as a function of the pH of the drug product (3.0–9.0) and the ionic strength (10 mM and 150 mM). The adsorption was most pronounced at a low ionic strength and the pH close to that of the isoelectric point of the protein. Moreover, adsorption at a high ionic strength was pH-independent, due to shielding of surface charges. The study was followed up with another article in which Dixit et al. [113], while using the same setup, assessed the influence of polysorbate 20 on Fc-fusion protein-silicon interaction. Interestingly, the authors observed a significant decrease in protein adsorption when the system was equilibrated with a polysorbate 20 solution prior to analysis. On the other hand, both polysorbate 20 and the Fc-fusion protein adsorbed at oil/water interface when mixed solution of the components was added into the QCM system. The influence of nonionic surfactants was further evaluated in the next article written by Dixit et al. [114]. The panel of the tested excipients was extended by polysorbate 80 and poloxamer 188. Nevertheless, the results were consistent with those for polysorbate 20: (i) pre-adsorbed surfactants inhibited protein adsorption to siliconized QCM crystal and (ii) when introduced into the system together with the surfactant and protein, they were adsorbed on the silicone oil layer. Based on the QCM and maximum bubble pressure technique results, the authors hypothesized that protein-surfactant binding might not be considered as a mechanism reducing adsorption of protein to the silicone oil. Similar conclusions regarding the mechanism of surfactant adsorption inhibition were made by Li et al. [115]. The authors utilized the QCM-D system to study the influence of polysorbate 80 and poloxamer 188 on abatacept-silicon oil interactions. In contrast to the previous publication by Dixit et al., however, polysorbate 80 turned out to be more effective than poloxamer 188 in prevention of protein adsorption. Polysorbate 80 superiority was explained in terms of an outcome of its rapid adsorption kinetics. Furthermore, Zheng et al. [116] applied the QCM-D system together with a micro-flow imaging (MFI) and Archimedes instrument to evaluate the role of polysorbate 80 in mitigation of particle formation in a protein drug product present in a prefilled syringe. The results showed that polysorbate 80 inhibited both the particle formation and protein adsorption to silicone oil. Moreover, their study proved that QCM-D can be successfully utilized as complementary to particle counting analytical methods in protein-silicone oil compatibility studies.

The QCM-D system has also been applied to study protein-protein interactions and viscosity of high-concentration (HC) formulations. Briefly, HC formulations typically contain a protein at concentrations of 100–200 mg/mL and are dedicated to subcutaneous injection. This form of protein drug presentation enables patients to self-administer the dose, without the need for hospitalization. On the other hand, protein formulations concentrated to such an extent are characterized by two potential challenges: (i) high protein aggregation tendency and (ii) high viscosity of the drug product [117]. Patel et al. [118] used the QCM-D system to evaluate the viscoelastic behavior of HC IgG_2_ solutions at different pH values. This was done via the calculation of the storage and loss moduli (G’ and G’’, respectively). The G’’/G’ ratio demonstrated the highest value for the pH 5.5 sample, thus indicating that the solution exhibited peak liquid-like behavior over the tested pH range. Additionally, Hartl et al. [119] studied the influence of pH and two excipients—citrate and histidine—on the viscoelastic properties of HC formulation of a monoclonal antibody (200 mg/mL). The authors fitted the QCM data to the Maxwell model. One of the fitting parameters—relaxation time τ—was correlated with other protein-protein interaction parameters, shear viscosity, second osmotic viral coefficient (B_22_), and a diffusion interaction parameter (k_D_). However, as opposed to self-interaction chromatography, static light scattering (B_22_ parameter) and DLS (k_D_ parameter) analyses, QCM analysis did not require sample dilution, and thus did not introduce a potential alternation in protein-protein interactions.

Finally, an example of using the QCM-D system in a study of clinical administration material compatibility was presented by Kapp et al. [120]. The authors studied adsorption of two IgG molecules diluted with 0.9% NaCl to a gold QCM-D sensor coated with propanethiol to simulate the hydrophobic surfaces encountered in infusion bags and intravenous lines. Both proteins were adsorbed in a comparable manner in the absence of polysorbate 80. Moreover, in both cases the adsorption was absent when the QCM-D sensor was pre-exposed to polysorbate 80.

The examples of QCM systems applied in formulation and primary packaging development studies are set out in Table 2. 

## 5. Application of the QCM in Drug Product Manufacturing Process Development

The protein and peptide parenteral drug product manufacturing process (otherwise known as ‘fill and finish’) consists of several stages. These may include, but are not limited to, drug substance thawing, formulation, mixing, sterile filtration, and filling into target primary packaging (vial, cartridge or prefillable syringe) followed by stoppering. The vials are then capped. If the product is intended to be presented as a lyophilizate, the filled vials, prior to capping, are loaded into the freeze dryer and the lyophilization cycle is executed. Finally, each product is subjected to visual inspection [106,121]. Designing the appropriate manufacturing process is of great importance for the quality of the final drug product. This emerges from the fact that during the fill and finish operations the molecule is subjected to shear forces and air/liquid interfaces which may lead to degradation [122]. The contact with incompatible solid/liquid interfaces is another potential risk factor during drug product manufacturing. Only a few articles describing the application of the QCM technique to process material compatibility studies have been published thus far. For instance, Oom et al. [123] utilized the QCM-D system to study the influence of molecule hydrophobicity, the presence of surfactant, pH, protein concentration, and a layer material (polystyrene, Teflon, Au, and silicon dioxide) on the adsorption propensity of the protein. Interestingly, although the presence of polysorbate 80 reduced the adsorption of the studied proteins to all surfaces, a significant portion of the more hydrophobic protein, mAb2, was still adsorbed to polystyrene and Teflon in the presence of this surfactant. In addition, the authors noticed that reversible adsorption to surfaces was stronger at higher protein concentrations. Furthermore, mAb2, a protein characterized by enhanced propensity to reversible self-association in solution, was also characterized by higher reversible adsorption as compared to mAb1. Kalonia et al. [124] investigated stainless steel surface-mediated particle formation as a function of protein concentration. Specifically, the authors used the QCM-D technique to evaluate the thickness, viscoelastic properties, and mass of the adsorbed protein layers. Protein primary layers deposited on QCM crystals during analysis of adsorption of HC formulations (≥50 mg/mL) were characterized by higher mass accumulation as compared to that of low concentration formulations. Interestingly, although a secondary protein layer (formed over the primary one) had thickness dependent on protein concentration, it remained liquid-like (two-layer Voigt model). The study was followed up by Defante et al. [125], where a similar stainless steel surface-mediated particle formation was studied for fusion protein. Overall, the results have shown that the QCM is a valuable supportive technique in root cause investigations for increased sub-visible particle content. The abovementioned drug product manufacturing process development studies are presented in Table 3.

## 6. Conclusions and Future Perspectives

The application of the QCM technique in the biopharmaceutical industry can be very useful. As presented above, it was successfully integrated into the analytical panel of particle formation-based root cause investigation, as well as primary packaging, formulation, and drug product process development. Depending on the study considered, the QCM analytical method was used to evaluate: (i) mass, (ii) thickness, (iii) viscoelastic properties, and (iv) reversibility of adsorbed protein layer. Significantly, the QCM method enabled analysis of HC protein formulations without the need of sample dilution. Furthermore, there has been a recent trend in automation of commercial QCM equipment. As an example, a fully automated QCM-D—*Q-Sense Pro* with autosampler module—was marketed enabling the user to conduct studies in a more reproducible manner with high efficiency [126]. Nevertheless, the QCM analyses still have some limitations, namely the lack of a standardization method for protein/peptide drug product development studies and a relatively long time of analysis (approximately 1 h). Another important problem with the QCM might be poor reproducibility of layer deposition on the surface of transducer, which can have a massive impact on the measurements with this technology [12,127] and might call for complementary techniques, such as microscopic characterization of obtained surfaces. However, to overcome this problem one can also acquire commercially available sensors [128]. It should be noted that although numerous scientific articles on QCM analysis of peptides can be found in the literature, none of them are dedicated to the pharmaceutical development of peptide drug products. To sum up, the QCM emerges as an analytical method with a significant potential to become one of the routine analyses in peptide and protein drug product development. However, further studies, especially ones dedicated to precision, accuracy, and robustness of the method are desirable in this field.

## Figures and Tables

**Table 1:** A selection of some QCM applications connected with formation of extracellular amyloid deposits or intracellular inclusions with amyloid-like characteristics.

Used Techniques	QCM Sensing Layer/System	Aggregating Protein/Peptide	Protein/Peptide Length	Disease/Application	Ref.
QCM, DLS, AFM	Ure2p covalently bonded to QCM	Ure2p protein, rate of fibril growth	354 or 359-	Fibril assembly	[78]
QCM, AFM, CD	Q-Sense E4, BiolinScientific, Sweden	Amylin aggregates and Au/g-C_3_N_4_	20–29	T2DM	[79]
QCM	5-MHz SiO_2_-coated QCM (Inficon, East Syracuse, NY, USA).	Aβ, binding interactions between SPBs and Aβ proteins	1–40	Alzheimer’s disease	[80]
QCM	QCM with Aβ_1–40_ intermediates	Aβ, rate of elongation monitoring	1–40	Alzheimer’s disease	[81]
QCM, AFM	QCM with short fibril segments	Insulin	21 + 30	Injection-localized amyloidosis	[82]
QCM-D	In situ multilayer amyloid deposition monitoring	Glucagon	29	Regulation of blood, treatment of severe hypoglycemia	[83]
TIRE, QCM	DE2 antibodies with PAH	Aβ in the direct immune reaction with monoclonal DE2 antibodies	1–16	Alzheimer’s disease	[84]
AFM, SPR, QCM-D	Q-Sense E1 BiolinScientific, Sweden	Aβ	1–42	Alzheimer’s disease	[85]
QCM, Simoa	Silica-coated crystals	Aβ, discrimination between monomers and oligomers	1–42	Alzheimer’s disease	[30]
QCM, AFM	-	Degradation of Aβ fibrils byphotoactive meso-tetra(4-sulfonatophenyl)porphyrin under UV irradiation	1–42	Alzheimer’s disease	[86]
QCM-D, Super Resolution Microscopy	QCM immobilized with fibrils	α-synuclein fibrils, secondary nucleation of monomers on fibril surface	140	Parkinson’s disease	[87]

Aβ—Amyloid- β protein/peptide, AFM—Atomic Force Microscopy, CD—Circular Dichroism, T2DM—Type 2 Diabetes Mellitus, XPS—X-ray Photoelectron Spectroscopy, SEM—Scanning Electron Microscope, CA—Contact Angle, ATR-FTIR—Attenuated Total Reflectance-shift Fourier Transform Infrared Spectroscopy, AL-BSA—Amyloid like-Bovine Serum Albumin, GA—Glutaraldehyde, SPBs—Supported Phospholipid Bilayers, TIRE—Total Internal Reflection Mode, PAH—poly(allylamine hydrochloride), Simoa—Single Molecular Array.

**Table 2:** Selection of QCM applications in formulation and primary packaging development-related studies.

Application	Method	Instrument	Molecule Type	Molecule Concentration	Ref.
Evaluation of HPβCD stabilizing properties	QCM-R	QCM200 + QCM25 (Stanford Research Systems)	IgG A, IgG B	0.1–1.0 mg/mL	[107]
Influence of pH and ionic strength on protein-silicone oil interactions	QCM-R	QCM200 (Stanford Research Systems)	Fc-fusion protein	0.001–1 mg/mL	[112]
Influence of polysorbate 20 on protein-silicone oil interactions	QCM-R	QCM200 (Stanford Research Systems)	Fc-fusion protein	0.1 mg/mL	[113]
Influence of polysorbate 20, polysorbate 80 and poloxamer 188 on protein-silicone oil interactions	QCM-R	QCM200 (Stanford Research Systems)	Fc-fusion protein	0.1 mg/mL	[114]
Influence of polysorbate 80 and poloxamer 188 on protein-silicone oil interactions	QCM-D	Q-Sense (Biolin Scientific Inc.)	Abatacept	1 and 10 mg/mL	[115]
Supplementary analysis during particle characterization studies (MFI and Archimedes) of protein drug product stored in prefilled syringes	QCM-D	Q-Sense (Biolin Scientific Inc.)	Therapeutic protein	0.3 mg/mL	[116]
Characterization of viscoelastic properties of HC protein formulation	QCM-D	Q-Sense (Biolin Scientific Inc.)	IgG_2_	70 mg/mL	[118]
Characterization of protein-protein interactions in HC protein formulation	QCM-I	QCM sensors (Suzhou SJ Biomaterials Co., Ltd.,) Network analyzer (N2PK design; Makarov Instruments)	Mab1	200 mg/mL	[119]
Clinical administration material compatibility study	QCM-D	Q-Sense (Biolin Scientific Inc.)	IgG_1_, IgG_2_	10 mg/mL	[120]

**Table 3:** Selection of the QCM system in drug product manufacturing process development-related studies.

Application	Method	Instrument	Molecule Type	Molecule Concentration	Ref.
Process material compatibility studies	QCM-D	Q-Sense (Biolin Scientific Inc.)	mAb 1, mAb 2	1 and 50 mg/mL	[123]
	QCM-D	Q-Sense (Biolin Scientific Inc.)	NISTmAb	0.1–100 mg/mL	[124]
	QCM-D	Q-Sense (Biolin Scientific Inc.)	Fc-fusion protein	0.1–110 mg/mL	[125]
